# Supplementary material for: Tumor Response and Its Impact on Treatment Failure in Rectal Cancer: Does Intensity of Neoadjuvant Treatment Matter?
Source: Cancers (Basel). 2024 Oct 30;16(21):3673. doi: 10.3390/cancers16213673 (PMC11544879; doi:10.3390/cancers16213673)
Supplement: Supplementary file 1 [file cancers-16-03673-s001.zip › cancers-3199973-supplementary.pdf]

Supplementary Online Content

Figure S1. Treatment schedules of the CAO/ARO/AIO-94, CAO/ARO/AIO-04 and CAO/ARO/AIO-12 trials

Figure S2. Study flow diagram of the present post-hoc analysis.

Table S1. Association of neoadjuvant treatment approach and reasons for treatment failure and median time to treatment failure

Table S2. Competing risk regression with death as competing risk (CI 95%) of clinical, surgical, pathological characteristics and risk of treatment failure according to neoadjuvant treatment approach

Figure S3. Cumulative incidence of locoregional recurrence/distant metastasis or death

Figure S4. Cumulative incidence of locoregional recurrence/distant metastasis stratified by response to neoadjuvant treatment

Figure S5. Cumulative incidence of locoregional recurrence/distant metastasis stratified by response to neoadjuvant treatment after 5-FU CRT

Figure S6. Cumulative incidence of locoregional recurrence/distant metastasis stratified by response to neoadjuvant treatment after 5-FU/Ox CRT

Figure S7. Cumulative incidence of locoregional recurrence/distant metastasis stratified by response to neoadjuvant treatment after TNT

Table S3. Competing Risk Regression with death as competing risk to assess statistical differences between treatment approaches stratified by TRG

Figure S8. Cumulative incidence of locoregional recurrence/distant metastasis stratified by TRG

Figure S9. Cumulative incidence of locoregional recurrence/distant metastasis stratified by TRG after 5-FU CRT

Figure S10. Cumulative incidence of locoregional recurrence/distant metastasis stratified by TRG after 5-FU/Ox CRT

Figure S11. Cumulative incidence of locoregional recurrence/distant metastasis stratified by TRG after TNT

Figure S12. Cumulative incidence of locoregional recurrence/distant metastasis according to TRG 0/1 after neoadjuvant 5-FU CRT, 5-FU/OX CRT or TNT

Figure S13. Cumulative incidence of locoregional recurrence/distant metastasis according to TRG 2/3 after neoadjuvant 5-FU CRT, 5-FU/OX CRT or TNT

Figure S14. Cumulative incidence of locoregional recurrence/distant metastasis according to TRG 4 after neoadjuvant 5-FU CRT, 5-FU/OX CRT or TNT

This supplementary material has been provided by the author to give readers additional information about their work.

Figure S1. Treatment schedules of the CAO/ARO/AIO-94, CAO/ARO/AIO-04 and CAO/ARO/AIO-12 trials

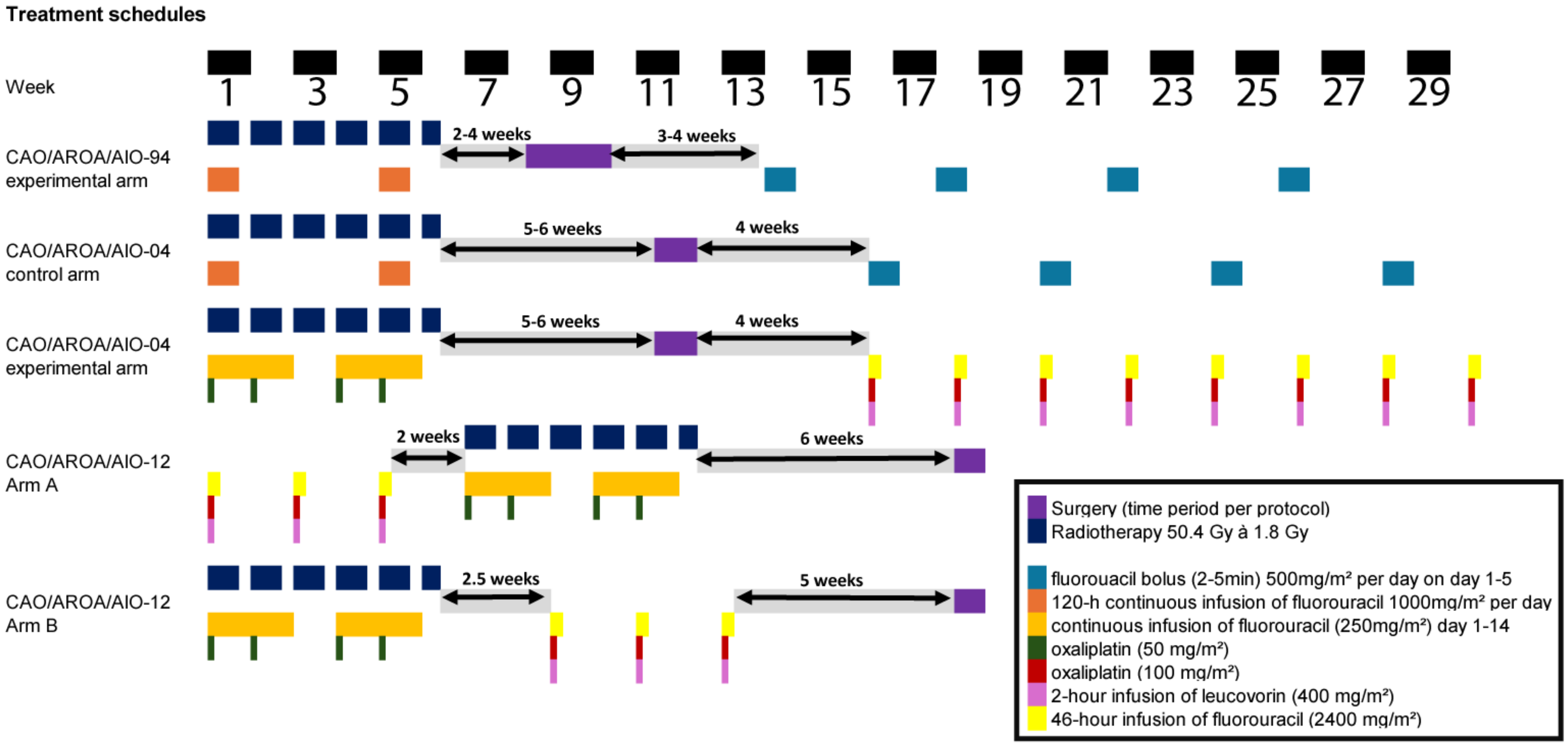

Figure S2. Study flow diagram of the present post-hoc analysis

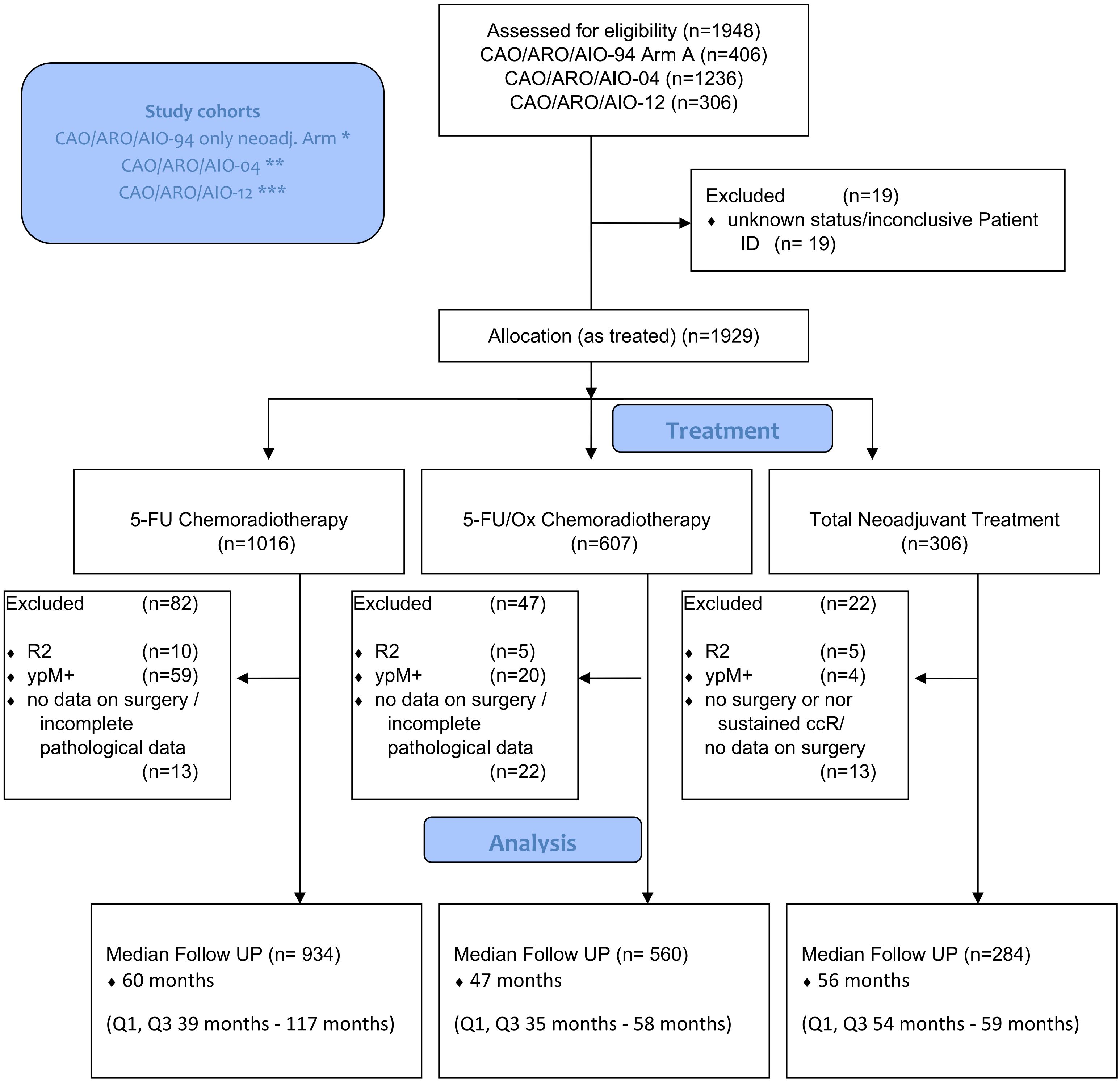

* Sauer R et al.; Preoperative versus postoperative chemoradiotherapy for locally advanced rectal cancer: results of the German CAO/ARO/AIO-94 randomized phase III trial after a median follow-up of 11 years. J Clin Oncol. 2012

** Rödel C et al.; German Rectal Cancer Study Group. Oxaliplatin added to fluorouracil-based preoperative chemoradiotherapy and postoperative chemotherapy of locally advanced rectal cancer (the German CAO/ARO/AIO-04 study): final results of the multicentre, open-label, randomised, phase 3 trial. Lancet Oncol. 2015

*** Fokas E et al.; Chemoradiotherapy Plus Induction or Consolidation Chemotherapy as Total Neoadjuvant Therapy for Patients With Locally Advanced Rectal Cancer: Long-term Results of the CAO/ARO/AIO-12 Randomized Clinical Trial. JAMA Oncol. 2022

Table S1. Association of neoadjuvant treatment approach and reasons for treatment failure and median time to treatment failure

Reason for Treatment failure	**Complete study cohort n=1778**	**5-FU CRT n = 934**	**5-FU/Ox CRT n=560**	**TNT n = 284**
Number of treatment failures (Median time to event [CI95%])	n= 378 (17months [15-19 months])	n=223 (16 months [14-21 months])	n=95 (19 months [16-23 months])	n=60 (16 months [14-24 months])
Number of Distant metastases as treatment failure defining event (Median time to event [CI95%])	n=320 (17 months [15-19 months])	n=187 (83.9%) (16 months [14-18 months])	n=83 (87.4%) (19 months [16-24 months])	n=50 (83.3%) (17 months [14-26 months])
Distant metastasis and locoregional recurrence as treatment failure defining event (Median time to event [CI95%])	N=21 (19 months [15-31 months])	n=13 (5.8%) (21 months [13-NA months])	n=6 (6.3%) (18 months [15-NA months])	n=2 (3.3%) 10 months [4-NA months])
Locoregional recurrence as treatment failure defining event (Median time to event [CI95%])	N=37 (17 months [13-28 months)]	n=23 (10.3%) (17 months [13-44 months])	n=6 (6.3%) (18 months (10-NA months])	n=8 (13.3%) (16 months [5-NA months])

Abbreviations: NA, not applicable

Table S2. Competing risk regression with death as competing risk (CI 95%) of clinical, surgical, pathological characteristics and risk of treatment failure according to neoadjuvant treatment approach

Characteristics	**Study cohort**	P-Value	**5-FU CRT**	P-Value	**5-FU/Ox CRT**	P-Value	**TNT**	P-Value
**Gender**								
Male	Reference		Reference		Reference		Reference	
Female	0.91 (0.73-1.14)	0.4	0.92 (0.68-1.24)	0.6	0.93 (0.60-1.47)	0.8	1.30 (0.77-2.20)	0.3
**Age**	1 (0.99-1.01)	0.4	1 (0.98-1.01)	0.5	1.01 (0.99-1.03)	0.5	0.98 (0.96-1.01)	0.3
**Tumor localisation**								
Low	Reference		Reference		Reference		Reference	
Intermediate	0.79 (0.63-0.97)	**0.03**	0.67 (0.50-0.88)	**0.01**	1.08 (0.71-1.65)	0.7	0.84 (0.49-1.44)	0.5
High	0.82 (0.57-1.18)	0.3	0.79 (0.50-1.26)	0.3	0.69 (0.31-1.56)	0.4	1.05 (0.45-2.44)	0.9
**cT**								
cT2	Reference		Reference		Reference		Reference	
cT3	1.78 (0.9-3.41)	0.08	1.46 (0.70-3.04)	0.3	1.72 (0.43-6.92)	0.5	23.5 (11.9-46.5)	**<0.001**
cT4	2.22 (1.08-4.57)	**0.03**	2.15 (0.92-5.00)	0.08	1.88 (0.39-9.19)	0.4	23.38 (9.6-56.9)	**<0.001**
**cN**								
cN0	Reference		Reference		Reference		Reference	
cN+	0.89 (0.71-1.11)	0.3	0.78 (0.59-1.02)	0.07	1.42 (0.85-2.37)	0.2	1.06 (0.43-2.62)	0.9
**Grading**								
G1	Reference		Reference		Reference		Reference	
G2	1.13 (0.65-1.97)	0.7	1.08 (0.51-2.31)	0.8	0.82 (0.33-2.00)	0.7	2.76 (0.37-20.5)	0.3
G3	1.83 (0.99-3.40)	0.05	1.90 (0.84-4.32)	**0.01**	1.61 (0.57-4.52)	0.4	0.71 (0.05-11.1)	0.8
**Type of surgery**								
Extirpation	Reference		Reference		Reference		Reference	
Anterior/deep anterior resection	0.60 (0.48-0.74)	**<0.001**	0.51 (0.38-0.67)	**<0.001**	0.77 (0.50-1.17)	0.2	0.72 (0.41-1.28)	0.3
Intersphincteric resection	0.66 (0.43-1.02)	0.06	0.50 (0.28-0.89)	**0.02**	0.66 (0.23-1.90)	0.4	1.34 (0.57-3.16)	0.5
other	0.65 (0.33-1.30)	0.2	1 (0.45-2.23)	0.90	0.25 (0.03-1.81)	0.2	0.40 (0.05-3.15)	0.4
**TME**								
complete	Reference				Reference		Reference	
no complete	1.33 (1.02-1.73)	**0.04**	1.44 (1.00-2.09)	0.05	2.15 (1.38-3.36)	**<0.001**	0.45 (0.2-1.03)	0.06
**Resection status**								
R0	Reference		Reference		Reference		Reference	
R1	1.61 (0.83-3.18)	0.2	3.24 (1.28-8.22)	**0.01**	0.53 (0.07-4.16)	0.6	1.29 (0.39-4.29)	0.7
**ypT**								
ypT0	Reference		Reference		Reference		Reference	
ypT1	1.83 (0.97-3.45)	0.06	1.84 (0.67-5.03)	0.2	2.13 (0.68-6.71)	0.2	1.76 (0.53-5.84)	0.4
ypT2	2.09 (1.32-3.31)	**0.01**	2.77 (1.33-5.77)	**0.01**	1.83 (0.77-4.39)	0.2	1.37 (0.55-3.39)	0.5

ypT3	4.74 (3.10-7.25)	**<0.001**	5.81 (2.88-11.7)	**<0.001**	4.66 (2.11-10.3)	**<0.001**	3.63 (1.71-7.69)	**<0.001**
ypT4	8.09 (4.41-14.8)	**<0.001**	11.1 (4.57-26.8)	**<0.001**	6.38 (1.67-24.4)	**0.01**	5.29 (1.59-17.6)	**0.01**
**ypN**								
ypN0	Reference		Reference		Reference		Reference	
ypN+	3.55 (2.90-4.35)	**<0.001**	3.62 (2.78-4.71)	**<0.001**	4.05 (2.70-6.07)	**<0.001**	2.74 (1.65-4.54)	**<0.001**
**ypL**								
ypL negative	Reference		Reference		Reference		Reference	
ypL positive	2.01 (1.58-2.56)	**<0.001**	2.03 (1.51-2.72)	**<0.001**	2.72 (1.64-4.50)	**<0.001**	0.61 (0.20-1.82)	0.4
**ypV**								
ypV negative	Reference		Reference		Reference		Reference	
ypV positive	2.13 (1.46-3.09)	**<0.001**	2.18 (1.37-3.48)	**<0.001**	1.08 (0.42-2.74)	0.9	1.11 (0.28-4.35)	0.9
**TRG**								
TRG 4	Reference		Reference		Reference		Reference	
TRG 2/3	3.10 (2.03-4.74)	**<0.001**	3.69 (1.83-7.44)	**<0.001**	3.16 (1.44-6.93)	**0.004**	2.47 (1.18-5.16)	**0.017**
TRG 0/1	4.84 (3.08-7.62)	**<0.001**	6.40 (3.11-13.2)	**<0.001**	4.12 (1.71-9.94)	**0.002**	2.63 (1.03-6.70)	**0.043**

Abbreviations: TRG, tumor regression grading to Dworak; ypV, post-neoadjuvant venous infiltration stage; ypL, post-neoadjuvant lymphatic vessel infiltration stage; ypN, post-neoadjuvant lymph node stage; ypT post-neoadjuvant tumor stage

Figure S3. Cumulative incidence of locoregional recurrence/distant metastasis or death

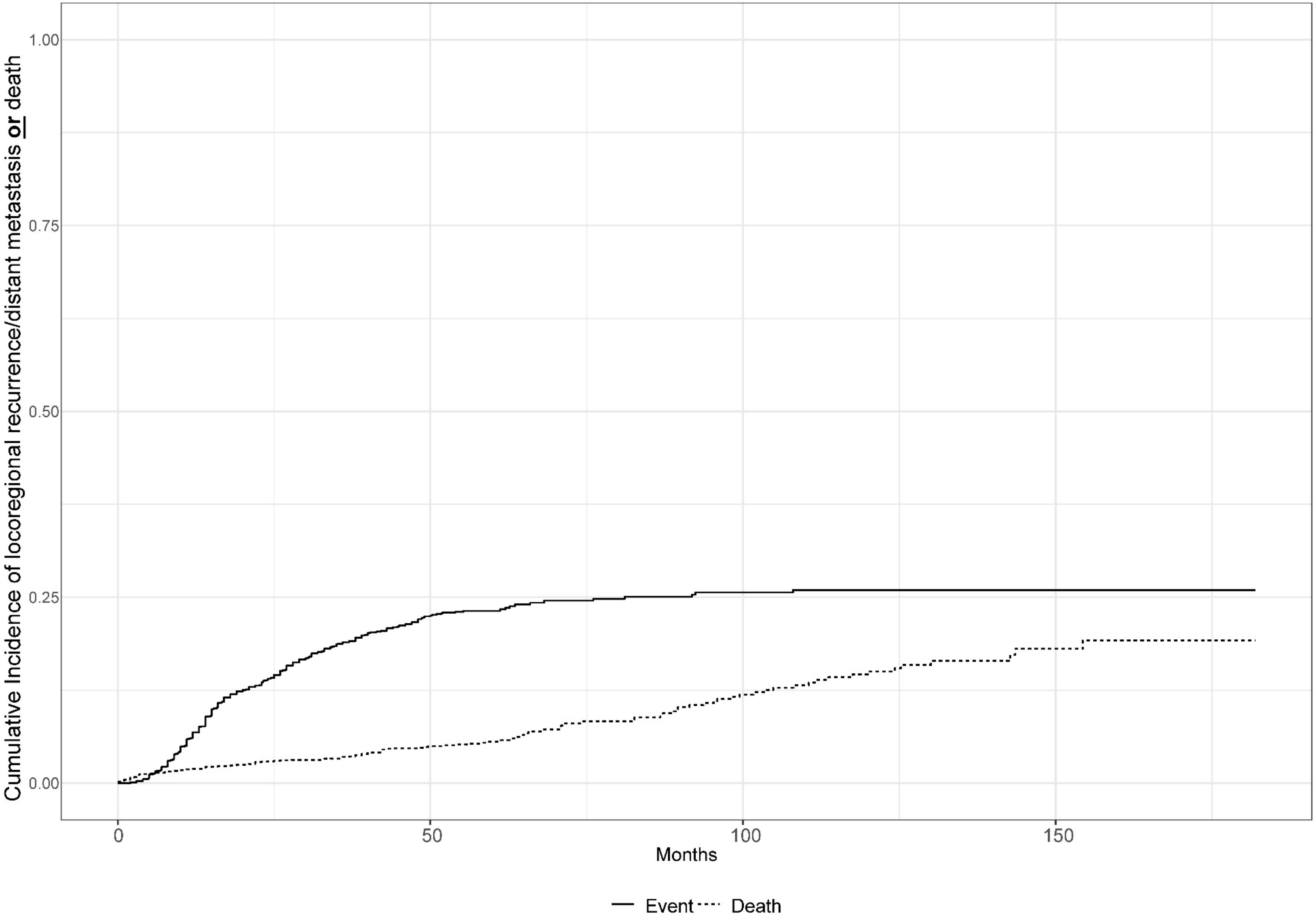

Figure S4. Cumulative incidence of locoregional recurrence/distant metastasis stratified by response to neoadjuvant treatment

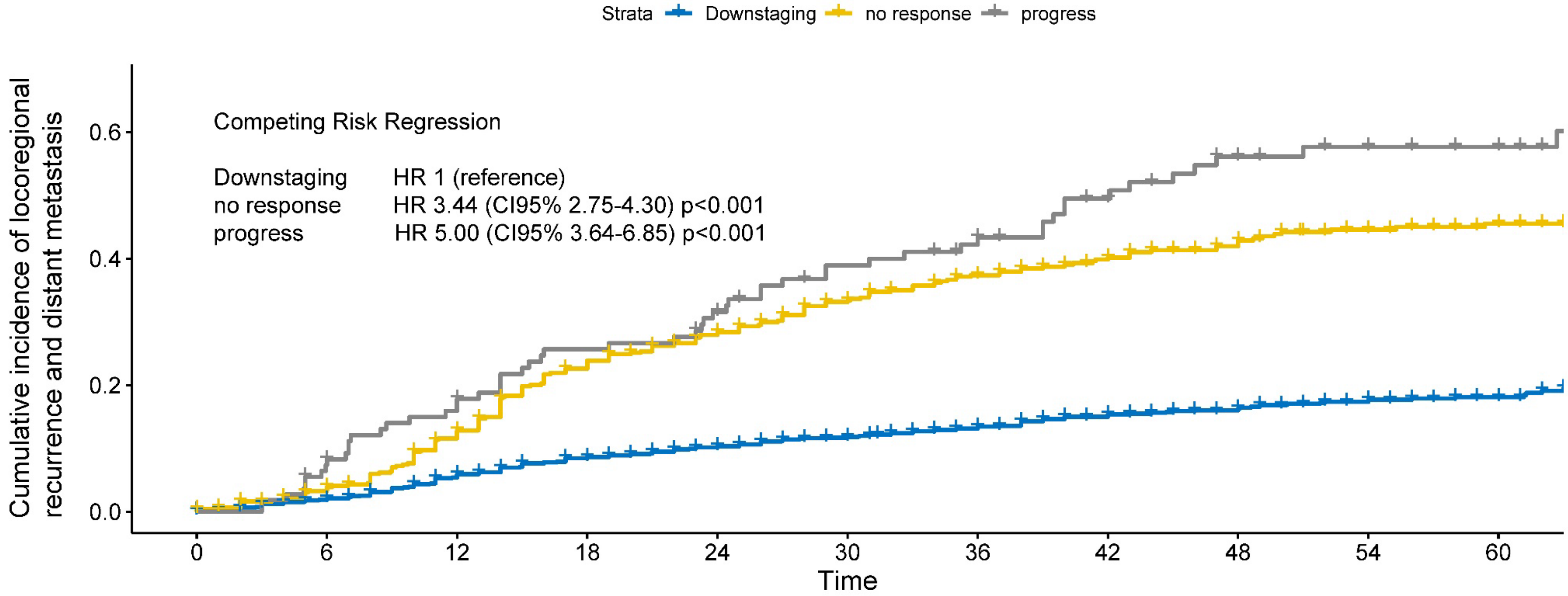

Number at risk (number of events)

Strata	0	6	12	18	24	30	36	42	48	54	60
Downstaging	1125 (2)	1075 (23)	1027 (65)	983 (94)	948 (112)	881 (127)	806 (143)	679 (159)	620 (166)	495 (175)	335 (177)
no response	494 (2)	469 (19)	423 (62)	364 (114)	328 (135)	291 (157)	254 (174)	211 (184)	190 (193)	148 (198)	107 (200)
progress	109 (0)	100 (9)	88 (19)	76 (27)	69 (33)	57 (40)	51 (44)	40 (49)	31 (54)	26 (55)	23 (55)

Time

Cumulative number of censoring

Strata	0	6	12	18	24	30	36	42	48	54	60
Downstaging	7	32	44	53	86	123	221	291	390	481	676
no response	1	12	18	22	40	49	84	103	131	153	208
progress	0	4	6	6	9	12	17	21	25	29	32

Time

Figure S5. Cumulative incidence of locoregional recurrence/distant metastasis stratified by response to neoadjuvant treatment after 5-FU CRT

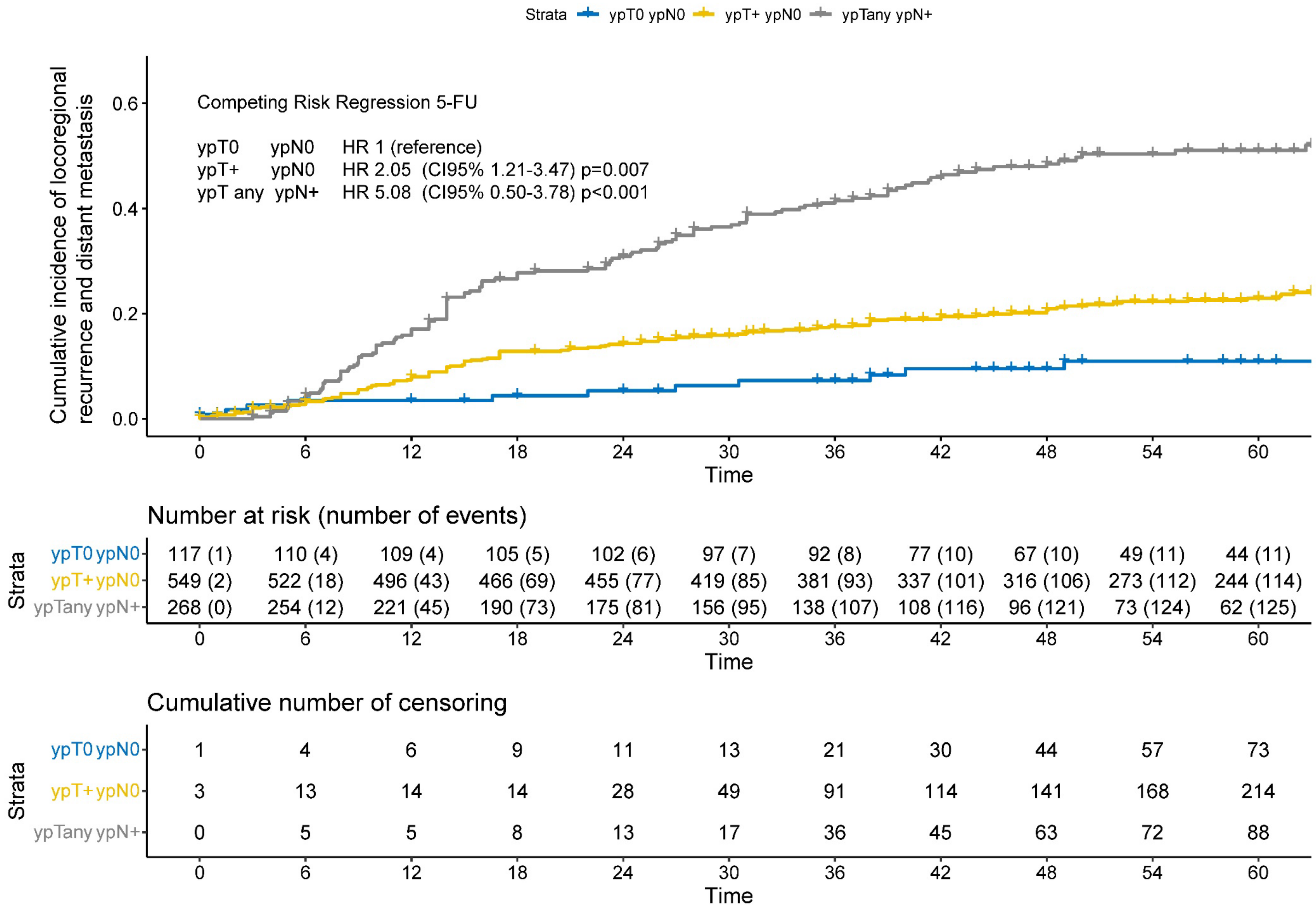

Figure S6. Cumulative incidence of locoregional recurrence/distant metastasis stratified by response to neoadjuvant treatment after 5-FU/Ox CRT

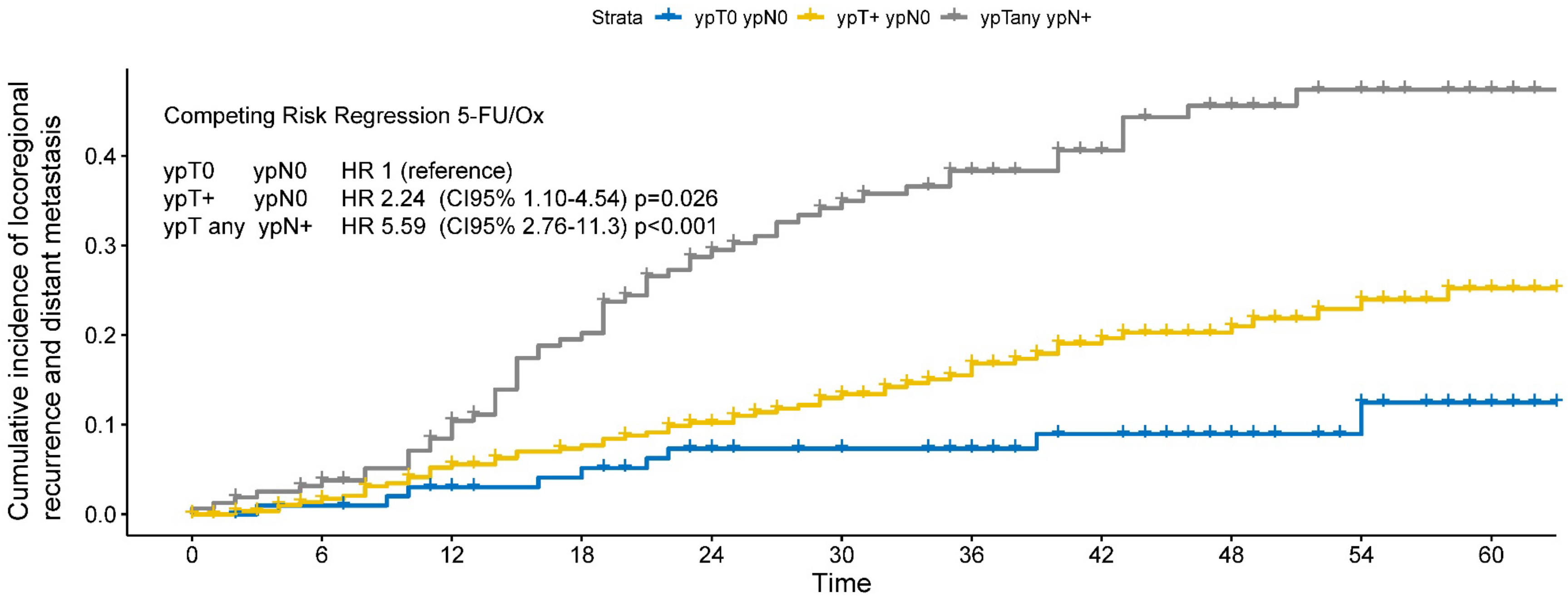

Number at risk (number of events)

Strata	0	6	12	18	24	30	36	42	48	54	60
ypT0 ypN0	104 (0)	99 (1)	94 (3)	90 (5)	84 (7)	80 (7)	73 (7)	54 (8)	45 (8)	26 (9)	15 (9)
ypT+ ypN0	297 (0)	285 (5)	269 (16)	259 (22)	242 (29)	214 (37)	188 (45)	135 (50)	116 (52)	72 (55)	50 (56)
ypTany ypN+	159 (1)	152 (6)	137 (16)	115 (30)	97 (43)	83 (50)	66 (54)	50 (56)	40 (60)	27 (61)	19 (61)

Time

Cumulative number of censoring

Strata	0	6	12	18	24	30	36	42	48	54	60
ypT0 ypN0	1	4	8	10	15	18	30	42	60	71	89
ypT+ ypN0	1	9	14	17	35	48	85	114	155	177	208
ypTany ypN+	0	7	14	15	24	28	45	55	64	72	86

Time

Figure S7. Cumulative incidence of locoregional recurrence/distant metastasis stratified by response to neoadjuvant treatment after TNT

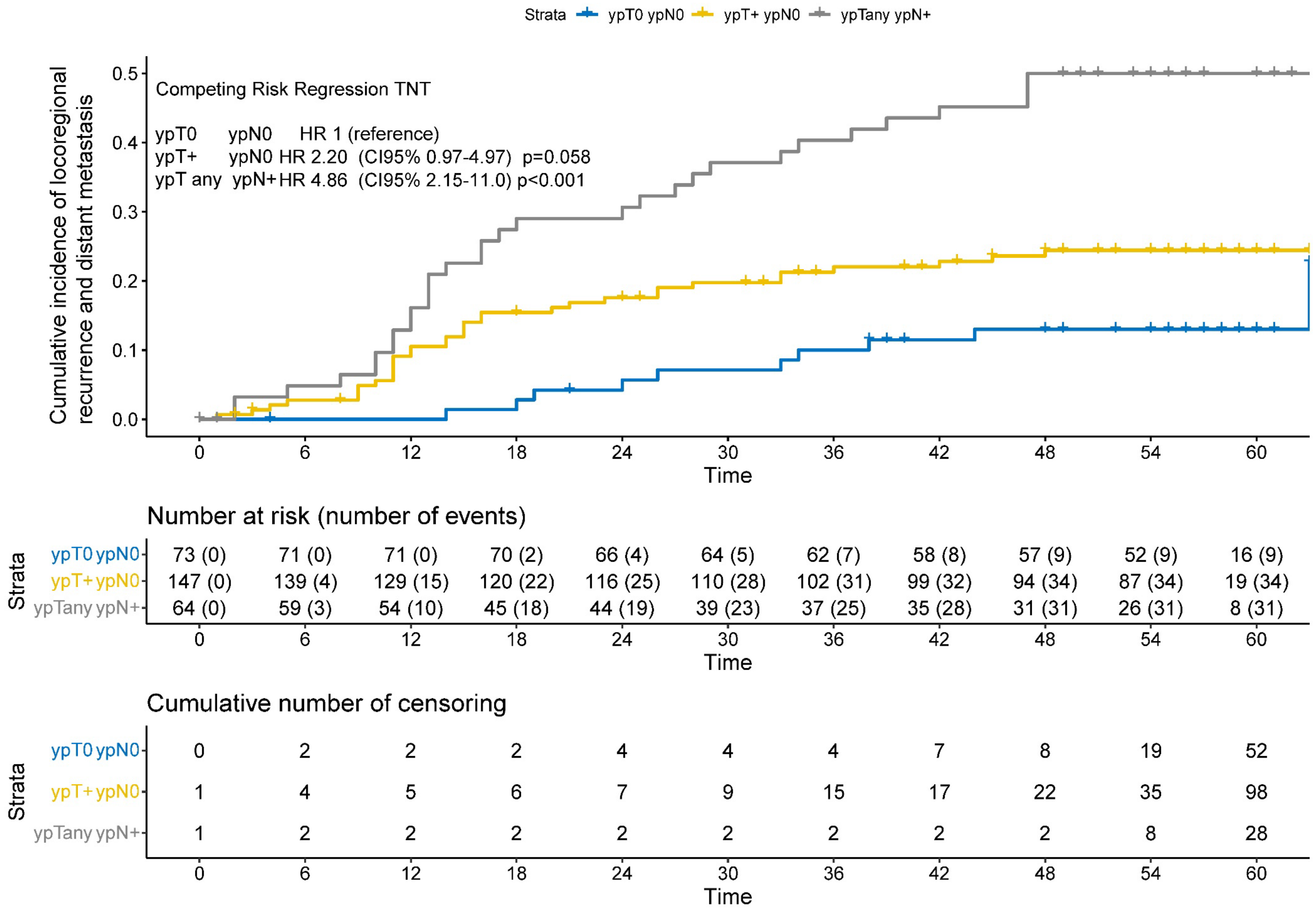

Table S3. Competing Risk Regression with death as competing risk to assess statistical differences between treatment approaches stratified by TRG

TRG	Treatment approach	HR (CI95%)	P-value
	5-FU CRT	Reference	
0/1	5-FU/Ox CRT	0.63 (0.38-1.06)	0.08
	TNT	0.66 (0.33-1.31)	0.23
	5-FU CRT	Reference	
2/3	5-FU/OX CRT	0.88 (0.66-1.17)	0.38
	TNT	1.07 (0.76-1.51)	0.71
	5-FU CRT	Reference	
4	5-FU/OX CRT	1.11 (0.41-3.02)	0.84
	TNT	1.64 (0.62-4.35)	0.32

Abbreviation: TRG, tumor regression grading to Dworak

Figure S8. Cumulative incidence of locoregional recurrence/distant metastasis stratified by TRG

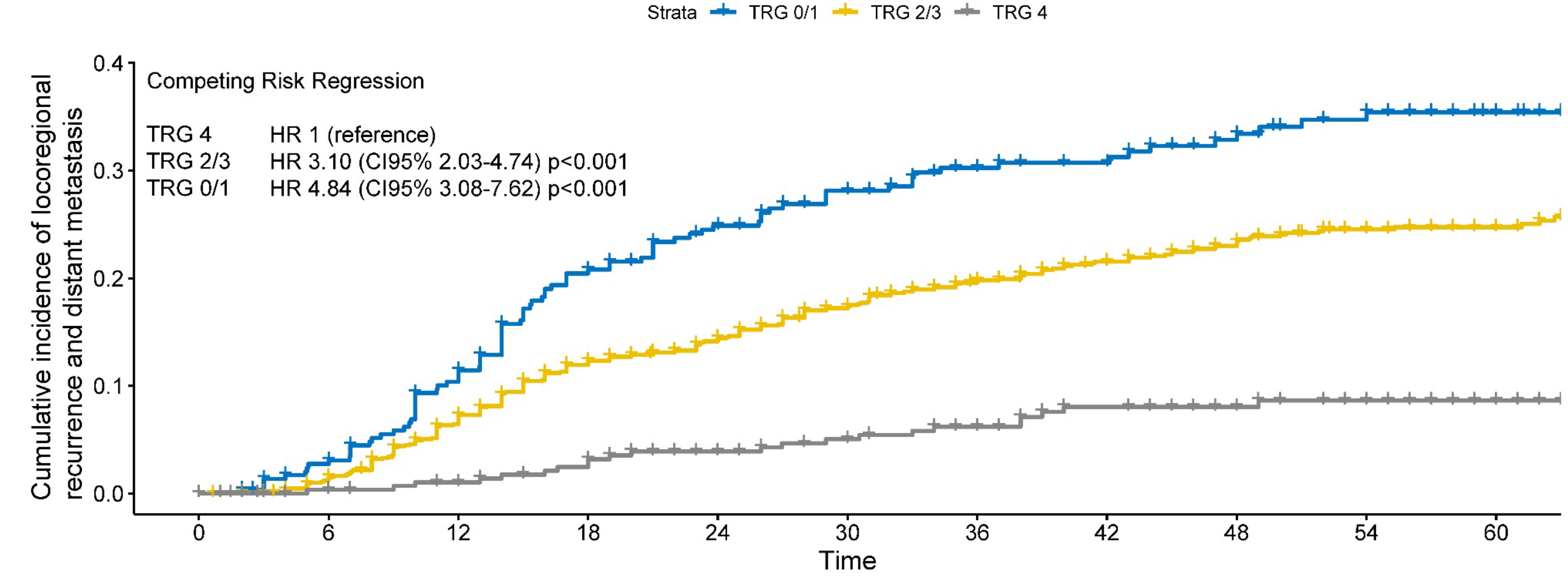

Number at risk (number of events)

Strata	0	6	12	18	24	30	36	42	48	54	60
TRG 0/1	300 (0)	282 (9)	255 (33)	220 (59)	201 (70)	175 (78)	156 (83)	135 (84)	119 (89)	96 (92)	74 (92)
TRG 2/3	1131 (0)	1082 (17)	1006 (79)	936 (133)	891 (155)	817 (185)	731 (208)	609 (222)	555 (236)	447 (243)	316 (244)
TRG 4	304 (0)	289 (1)	283 (3)	273 (9)	259 (11)	247 (14)	232 (17)	191 (21)	170 (21)	126 (22)	73 (22)

Time

Cumulative number of censoring

Strata	0	6	12	18	24	30	36	42	48	54	60
TRG 0/1	3	11	19	23	38	49	71	82	102	115	146
TRG 2/3	5	45	59	67	101	135	237	309	388	461	620
TRG 4	3	15	21	26	39	44	66	92	128	165	233

Time

Figure S9. Cumulative incidence of locoregional recurrence/distant metastasis stratified by TRG after 5-FU CRT

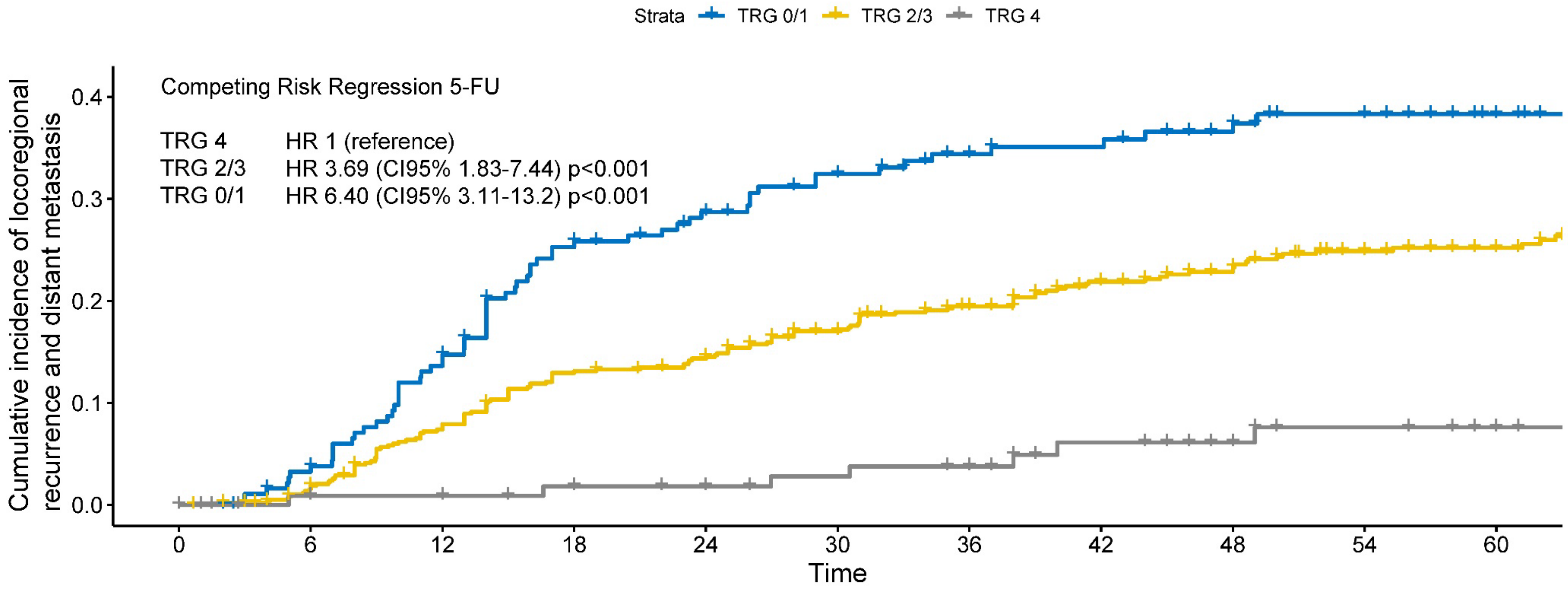

Number at risk (number of events)

Strata	0	6	12	18	24	30	36	42	48	54	60
TRG 0/1	188 (0)	178 (7)	158 (27)	134 (47)	122 (52)	108 (58)	99 (61)	88 (62)	78 (65)	65 (66)	57 (66)
TRG 2/3	604 (0)	575 (11)	536 (46)	501 (76)	487 (84)	449 (98)	404 (111)	344 (122)	321 (128)	269 (134)	237 (135)
TRG 4	119 (0)	112 (1)	111 (1)	107 (2)	104 (2)	99 (3)	94 (4)	77 (6)	67 (6)	49 (7)	44 (7)

Time

Cumulative number of censoring

Strata	0	6	12	18	24	30	36	42	48	54	60
TRG 0/1	1	5	6	9	20	23	34	38	51	58	69
TRG 2/3	3	23	25	28	38	60	110	142	174	205	258
TRG 4	2	7	9	12	15	17	26	36	50	63	79

Time

Figure S10. Cumulative incidence of locoregional recurrence/distant metastasis stratified by TRG after 5-FU/Ox CRT

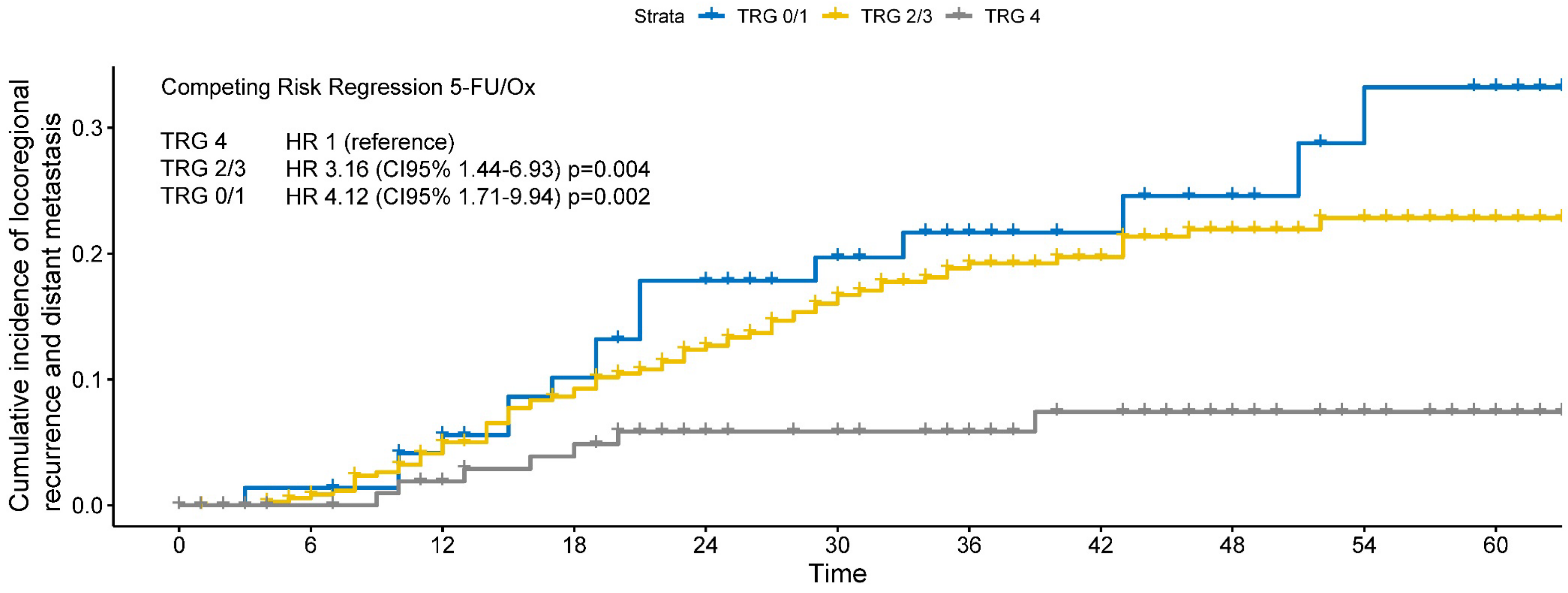

Number at risk (number of events)

Strata	0	6	12	18	24	30	36	42	48	54	60
TRG 0/1	76 (0)	72 (1)	67 (4)	59 (7)	53 (12)	43 (13)	37 (14)	27 (14)	24 (15)	16 (17)	13 (17)
TRG 2/3	357 (0)	345 (3)	320 (17)	300 (31)	273 (42)	245 (54)	210 (61)	153 (62)	128 (66)	82 (67)	55 (67)
TRG 4	113 (0)	107 (0)	102 (2)	97 (5)	90 (6)	86 (6)	78 (6)	58 (7)	48 (7)	26 (7)	15 (7)

Time

Cumulative number of censoring

Strata	0	6	12	18	24	30	36	42	48	54	60
TRG 0/1	1	3	9	10	14	21	29	35	40	44	52
TRG 2/3	1	17	26	28	51	61	109	146	190	215	253
TRG 4	1	6	10	12	19	22	35	48	68	82	100

Time

Figure S11. Cumulative incidence of locoregional recurrence/distant metastasis stratified by TRG after TNT

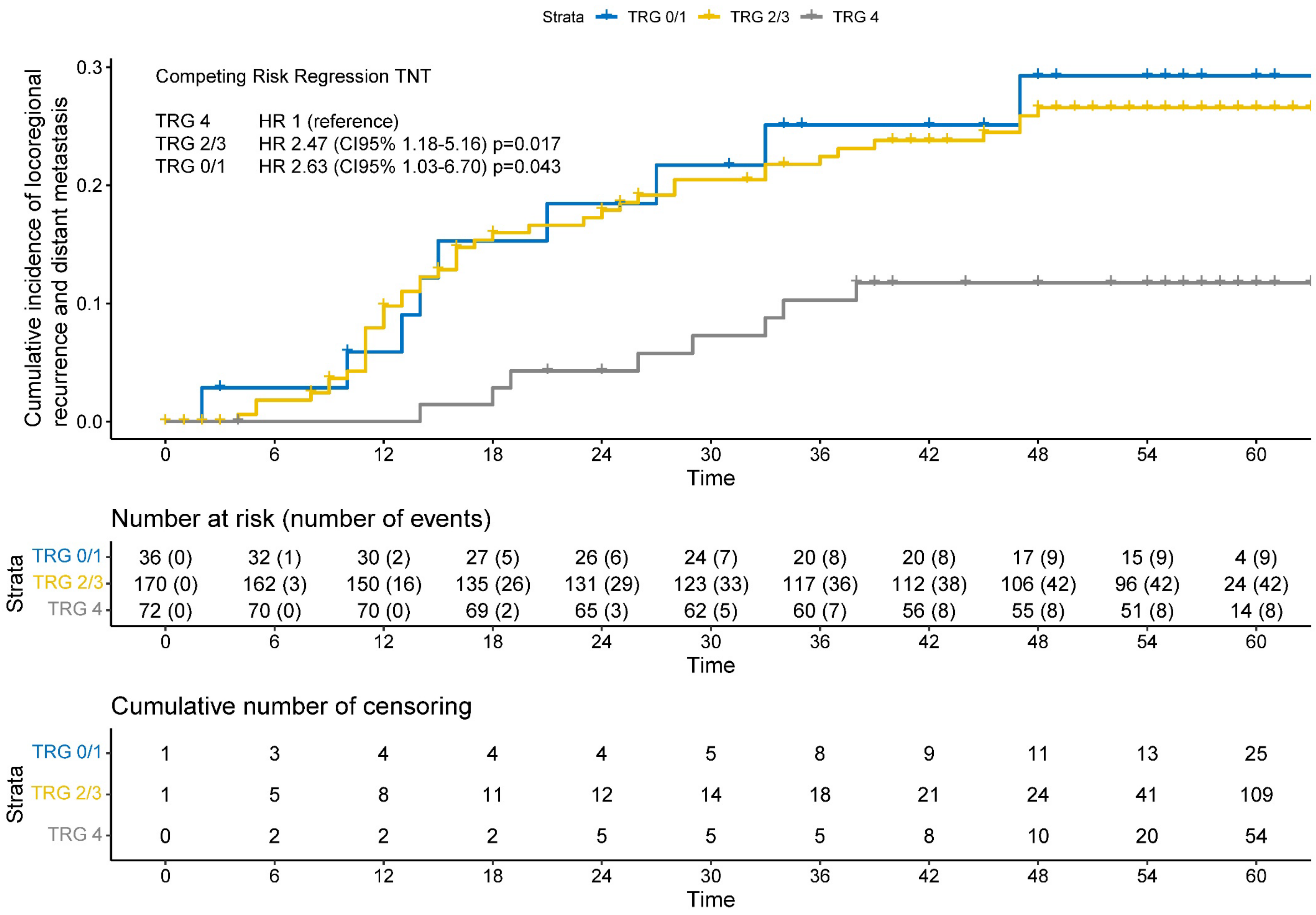

Figure S12. Cumulative incidence of locoregional recurrence/distant metastasis according to TRG 0/1 after neoadjuvant 5-FU CRT, 5-FU/OX CRT or TNT

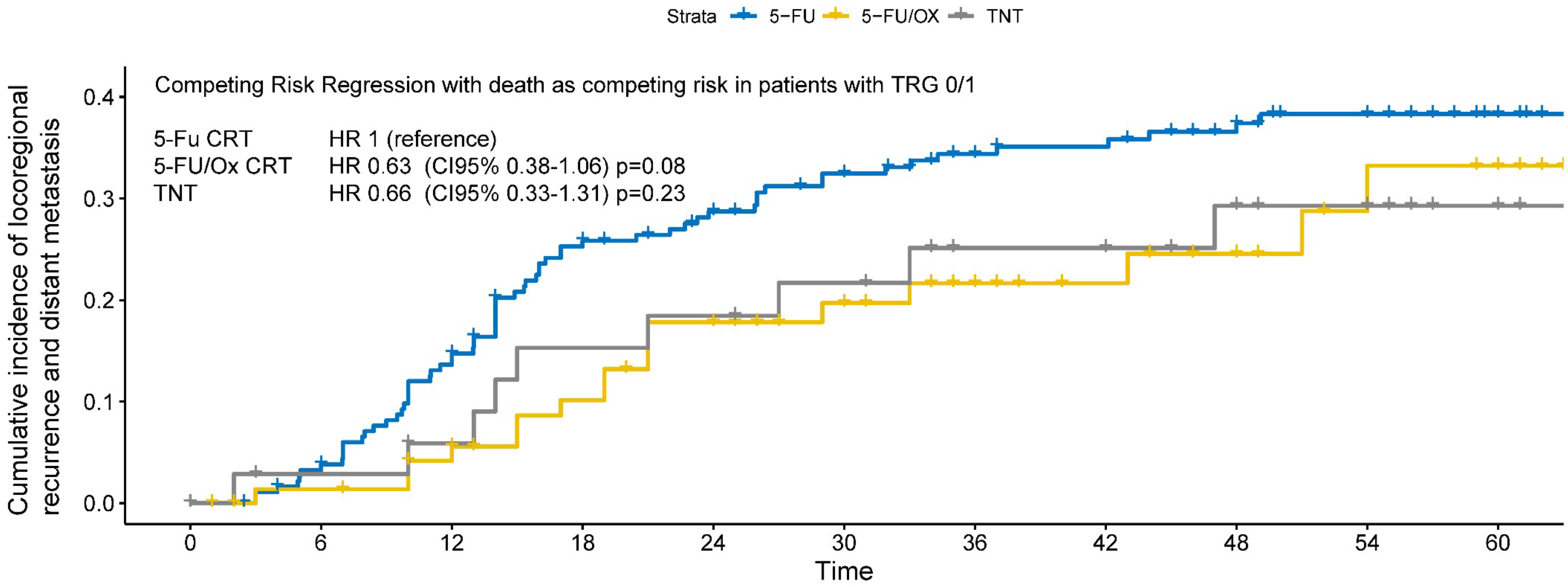

Number at risk (number of events)

	0	6	12	18	24	30	36	42	48	54	60
5−FUCRT	188 (0)	178 (7)	158 (27)	134 (47)	122 (52)	108 (58)	99 (61)	88 (62)	78 (65)	65 (66)	57 (66)
5−FU/OxCRT	76 (0)	72 (1)	67 (4)	59 (7)	53 (12)	43 (13)	37 (14)	27 (14)	24 (15)	16 (17)	13 (17)
TNT	36 (0)	32 (1)	30 (2)	27 (5)	26 (6)	24 (7)	20 (8)	20 (8)	17 (9)	15 (9)	4 (9)

Time

Cumulative number of censoring

	0	6	12	18	24	30	36	42	48	54	60
5−FUCRT	1	5	6	9	20	23	34	38	51	58	69
5−FU/OxCRT	1	3	9	10	14	21	29	35	40	44	52
TNT	1	3	4	4	4	5	8	9	11	13	25

Time

Figure S13. Cumulative incidence of locoregional recurrence/distant metastasis according to TRG 2/3 after neoadjuvant 5-FU CRT, 5-FU/OX CRT or TNT

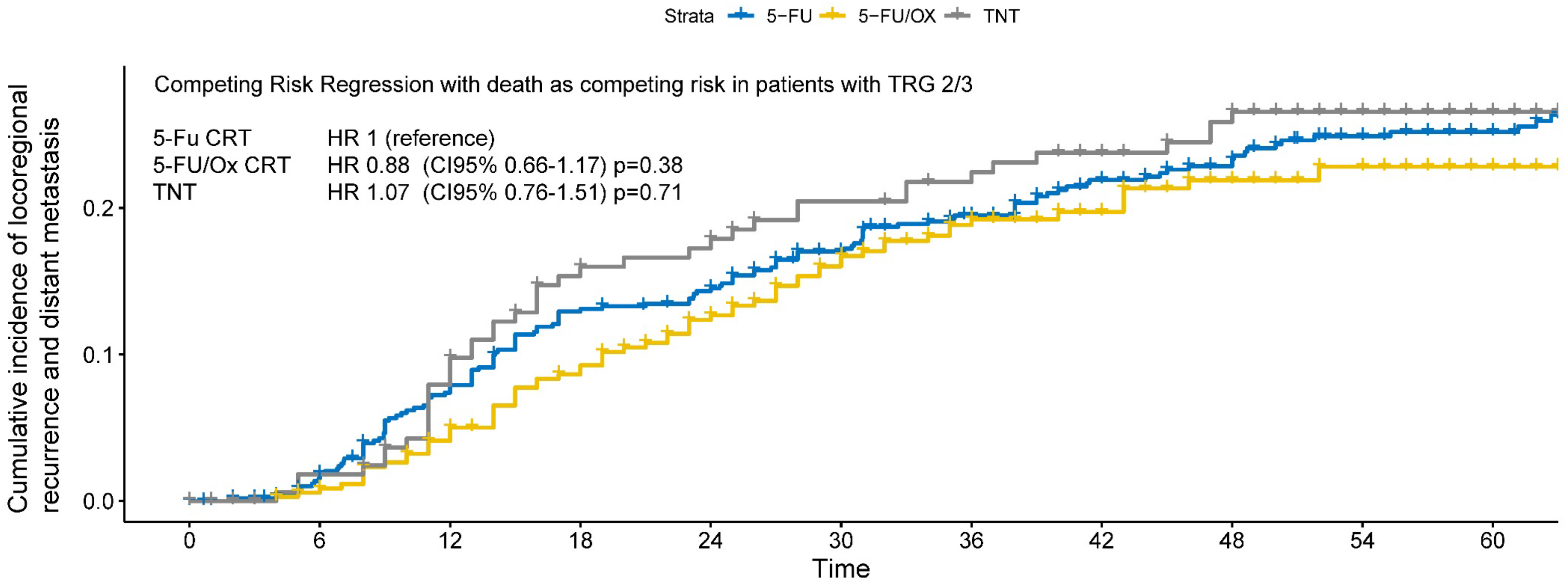

Number at risk (number of events)

	0	6	12	18	24	30	36	42	48	54	60
5−FUCRT	604 (0)	575 (11)	536 (46)	501 (76)	487 (84)	449 (98)	404 (111)	344 (122)	321 (128)	269 (134)	237 (135)
5−FU/OxCRT	357 (0)	345 (3)	320 (17)	300 (31)	273 (42)	245 (54)	210 (61)	153 (62)	128 (66)	82 (67)	55 (67)
TNT	170 (0)	162 (3)	150 (16)	135 (26)	131 (29)	123 (33)	117 (36)	112 (38)	106 (42)	96 (42)	24 (42)

Time

Cumulative number of censoring

	0	6	12	18	24	30	36	42	48	54	60
5−FUCRT	3	23	25	28	38	60	110	142	174	205	258
5−FU/OxCRT	1	17	26	28	51	61	109	146	190	215	253
TNT	1	5	8	11	12	14	18	21	24	41	109

Time

Figure S14. Cumulative incidence of locoregional recurrence/distant metastasis according to TRG 4 after neoadjuvant 5-FU CRT, 5-FU/OX CRT or TNT

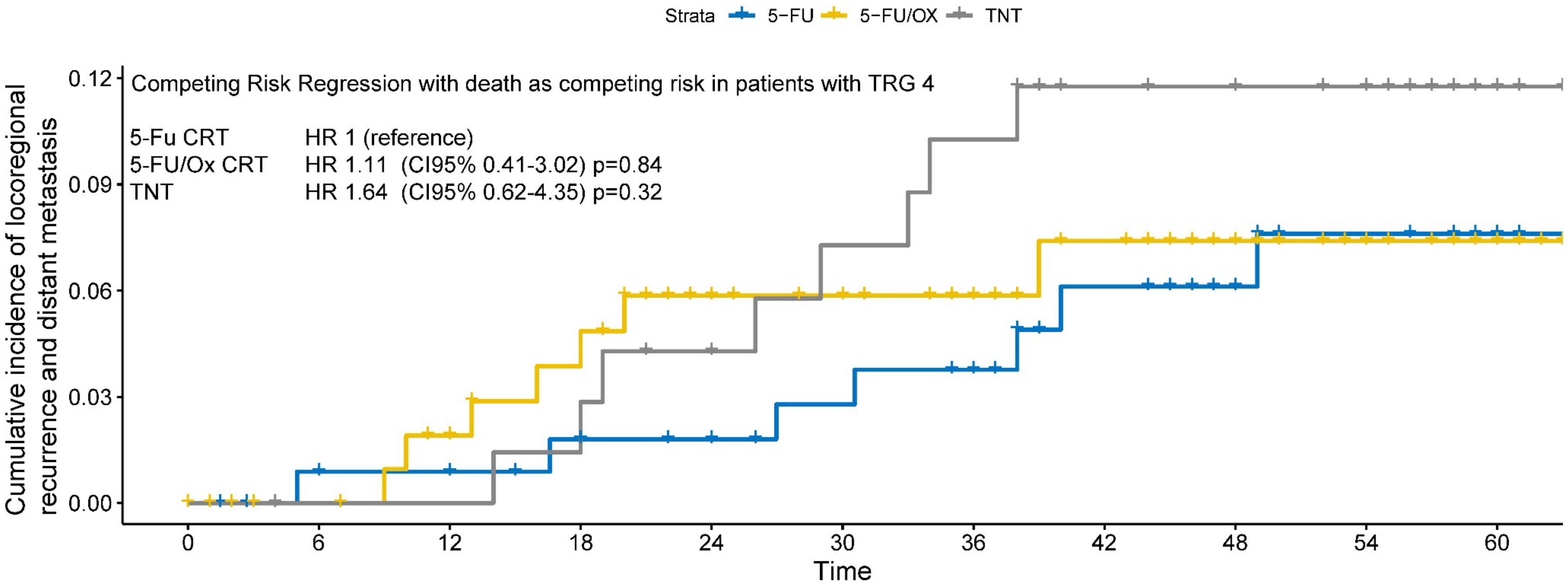

Number at risk (number of events)

Time	0	6	12	18	24	30	36	42	48	54	60
5−FUCRT	119 (0)	112 (1)	111 (1)	107 (2)	104 (2)	99 (3)	94 (4)	77 (6)	67 (6)	49 (7)	44 (7)
5−FU/OxCRT	113 (0)	107 (0)	102 (2)	97 (5)	90 (6)	86 (6)	78 (6)	58 (7)	48 (7)	26 (7)	15 (7)
TNT	72 (0)	70 (0)	70 (0)	69 (2)	65 (3)	62 (5)	60 (7)	56 (8)	55 (8)	51 (8)	14 (8)

Cumulative number of censoring

Time	0	6	12	18	24	30	36	42	48	54	60
5−FUCRT	2	7	9	12	15	17	26	36	50	63	79
5−FU/OxCRT	1	6	10	12	19	22	35	48	68	82	100
TNT	0	2	2	2	5	5	5	8	10	20	54
